# The Causal Effects of Insomnia on Bipolar Disorder, Depression, and Schizophrenia: A Two-Sample Mendelian Randomization Study

**DOI:** 10.3389/fgene.2021.763259

**Published:** 2021-10-11

**Authors:** Peng Huang, Yixin Zou, Xingyu Zhang, Xiangyu Ye, Yidi Wang, Rongbin Yu, Sheng Yang

**Affiliations:** ^1^ Department of Epidemiology, Center for Global Health, School of Public Health, Nanjing Medical University, Nanjing, China; ^2^ Thomas E. Starzl Transplantation Institute, University of Pittsburgh Medical Center, University of Pittsburgh, Pittsburgh, PA, United States; ^3^ Department of Biostatistics, Center for Global Health, School of Public Health, Nanjing Medical University, Nanjing, China

**Keywords:** insomnia, bipolar disorder, depression, schizophrenia, two-sample mendelian randomization, genome-wide association study

## Abstract

Psychiatric disorder, including bipolar disorder (BD), major depression (MDD), and schizophrenia (SCZ), affects millions of persons around the world. Understanding the disease causal mechanism underlying the three diseases and identifying the modifiable risk factors for them hold the key for the development of effective preventative and treatment strategies. We used a two-sample Mendelian randomization method to assess the causal effect of insomnia on the risk of BD, MDD, and SCZ in a European population. We collected one dataset of insomnia, three of BD, one of MDD, and three of SCZ and performed a meta-analysis for each trait, further verifying the analysis through extensive complementarity and sensitivity analysis. Among the three psychiatric disorders, we found that only insomnia is causally associated with MDD and that higher insomnia increases the risk of MDD. Specifically, the odds ratio of MDD increase of insomnia is estimated to be 1.408 [95% confidence interval (CI): 1.210–1.640, *p* = 1.03E-05] in the European population. The identified causal relationship between insomnia and MDD is robust with respect to the choice of statistical methods and is validated through extensive sensitivity analyses that guard against various model assumption violations. Our results provide new evidence to support the causal effect of insomnia on MDD and pave ways for reducing the psychiatric disorder burden.

## Introduction

Insomnia disorder is predominantly characterized by dissatisfaction with sleep duration or quality and difficulties in initiating or maintaining sleep (Morin et al., 2015; Winkelman, 2015). Most cross-sectional and longitudinal studies have also shown that insomnia increases the risks of acute myocardial infarction and coronary heart disease, heart failure, hypertension, diabetes, and death, particularly when insomnia is accompanied by a short total sleep duration (<6 h per night) (Chen et al., 2013; Morin et al., 2015; Parthasarathy et al., 2015; Grandner et al., 2016; Javaheri and Redline, 2017; Bertisch et al., 2018; Dong and Yang, 2019). Emerging evidence show that insomnia associates to both incident and some recurrent psychiatric disorders, including major depression disorder (MDD), anxiety disorder, substance use problems, and suicidality. In addition, a wide range of sociodemographic correlates of insomnia have been identified and include advanced age, female sex, low socioeconomic status, unemployment, and psychological distress. Although insomnia results from environmental factors, it is, in part, attributable to genetic factors (Winkelman, 2015).

The generation and development of psychiatric disorders are influenced by genetic and environmental factors (Sklar et al., 2011; Nagel et al., 2018; Ruderfer et al., 2018; Peng et al., 2020; Peng et al., 2021a; Peng et al., 2021b). For genetic factors, based on genome-wide association analysis (GWAS), Purcell *et al.* implicate the major histocompatibility complex, constructed a polygenic risk score (PRS) of schizophrenia (SCZ) and verified that the PRS also predicted bipolar disorder (BD) (Purcell et al., 2009). For environmental factors, using a case–control study, Palagini *et al.* found that insomnia played a mediating role between early life stress and the clinical manifestations of BD, and assessing the evolution of insomnia symptoms can provide a basis for the characteristics and treatment strategies of BD (Palagini et al., 2021). In addition, the result of longitudinal epidemiological studies shows that sleep disturbances and insomnia increase the risk of MDD after 1–3 years (Riemann and Voderholzer, 2003; Franzen and Buysse, 2008). Studies have also shown that up to 80% of patients with SCZ report symptoms of insomnia (Stummer et al., 2018). Sleep disorders have been shown to increase the risk of cognitive impairment and recurrence in patients with schizophrenia (Stummer et al., 2018). However, all these findings are summarized from either observational studies or pilot randomized controlled trials and prone to selection bias, especially unobserved confounding factors—that is, correlation cannot be simply equal to causal association. It is essential and urgent to further investigate the causal association between insomnia and psychiatric disorders, including BD, MDD, and SCZ.

Based on Mendel’s law of inheritance—that is, parental alleles are randomly assigned to offspring—Mendelian randomization (MR), an advanced statistical method, treats single-nucleotide polymorphism (SNP) as an instrumental variable (IV) to adjust the effect of confounders and identifies the causal relationship between two traits (Davey Smith and Hemani, 2014; Paternoster et al., 2017). Then, when we regarded SNPs both with association to insomnia and without association to psychiatric disorders as IVs, MR can establish the causal relationship between insomnia and psychiatric disorders. Because genetic variants are fixed at conception and cannot be modified subsequently, MR can overcome a possible reverse causation. MR assumes that if insomnia causes psychiatric disorders, SNP related to insomnia causes psychiatric disorders through the insomnia pathway. Emerging large-scale GWAS of insomnia and psychiatric disorders gives us opportunities to use MR to study the causal relationship between them (Sleiman and Grant, 2010).

In the present study, our main aim is to investigate the causal relationship between insomnia and three psychiatric disorders (BD, MDD, and SCZ) in a European ancestry. To achieve the aim, we used the summary statistics of eight datasets (including 386,533 samples of insomnia and 719,027 samples of three psychiatric disorders) to perform a series of two-sample MR to comprehensively elucidate the potential causal association between insomnia and BD, MDD, and SCZ. In addition, to ensure the validity of the results of MR, we performed three sensitivity analyses, including heterogeneity test, pleiotropy test, and leave-one-out (LOO) test, and reverse-direction MR analyses (Zeng et al., 2019; Zeng and Zhou, 2019; Gormley et al., 2021).

## Materials and Methods

### GWAS Meta-Analysis

We collected eight datasets of insomnia and three psychiatric disorders from the GWAS-ATLAS (https://atlas.ctglab.nl/) (Tian et al., 2020), including one insomnia dataset (Jansen et al., 2019), three BD datasets (Smith et al., 2009; Hou et al., 2016; Ruderfer et al., 2018), one MDD dataset (Howard et al., 2019), and three SCZ datasets (Manolio et al., 2007; Ripke et al., 2013; Pardiñas et al., 2018). The insomnia summary statistics was estimated from the UK Biobank datasets with 386,533 individuals (Prev. = 0.283). The MDD summary statistics was estimated from the UK Biobank datasets with 500,199 individuals (Prev. = 0.341). The three BD summary statistics had 34,950 individuals (Prev. = 0.219), 2,035 individuals (Prev. = 0.492), and 41,653 individuals (Prev. = 0.483), repectively. The three SCZ summary statistics had 32,143 individuals (Prev. = 0.430), 2,729 individuals (Prev. = 0.495), and 105,318 individuals (Prev. = 0.386), repectively. The three studies for BD and MDD were without any overlap individuals. All summary statistics were estimated in the European ancestry. Then, we filtered out SNPs 1) with INFO <0.6, 2) with MAF <0.01, 3) with palindromic allele, and 4) whose OR was larger or smaller than mean ±3 SD. Finally, we obtained 7,213,582, 9,018,454, 7,743,682, and 8,679,614 SNPs for the four traits. Details of the meta-dataset and the three datasets for BD and SCZ are shown in Table 1 and Supplementary Table S1.

**TABLE 1:** Summary of the meta-datasets for four traits.

Trait	*N*_SNP_	*N*_sample_	Prev	$h_{O}^{2}$	$h_{L}^{2}$	$\lambda_{GC}$	Intcp
Insomnia	7,213,582	386,533	0.283	0.046	0.082	1.310	1.015
BD	9,018,454	78,638	0.366	0.405	0.286	1.421	1.080
MDD	7,743,682	500,199	0.341	0.060	0.067	1.453	1.00
SCZ	8,679,614	140,190	0.399	0.295	0.170	1.637	1.044

Furthermore, to obtain an accurate and robust estimation for each variant, we performed GWAS meta-analysis for each trait using METAL (v2011-03-25) (Willer et al., 2010). To control the population stratification, we set the option *GENOMICCONTROL* to on. In addition, we used Linkage Disequilibrium SCore regression (LDSC) (v1.0.1) to estimate both the observed and liability observed heritability (*h* ^2^) for each trait. We set the population prevalence (--*pop-prev*) for the four traits to 0.300, 0.020, 0.086, and 0.010 to estimate liability heritability, respectively (Ayuso-Mateos et al., 2001; Roth, 2007; Di Luca et al., 2011). We also estimated the genetic correlation (*R*_*g*_) between them in the GWAS analysis results (Tylee et al., 2018).

### IV Selection

**FIGURE 1:**
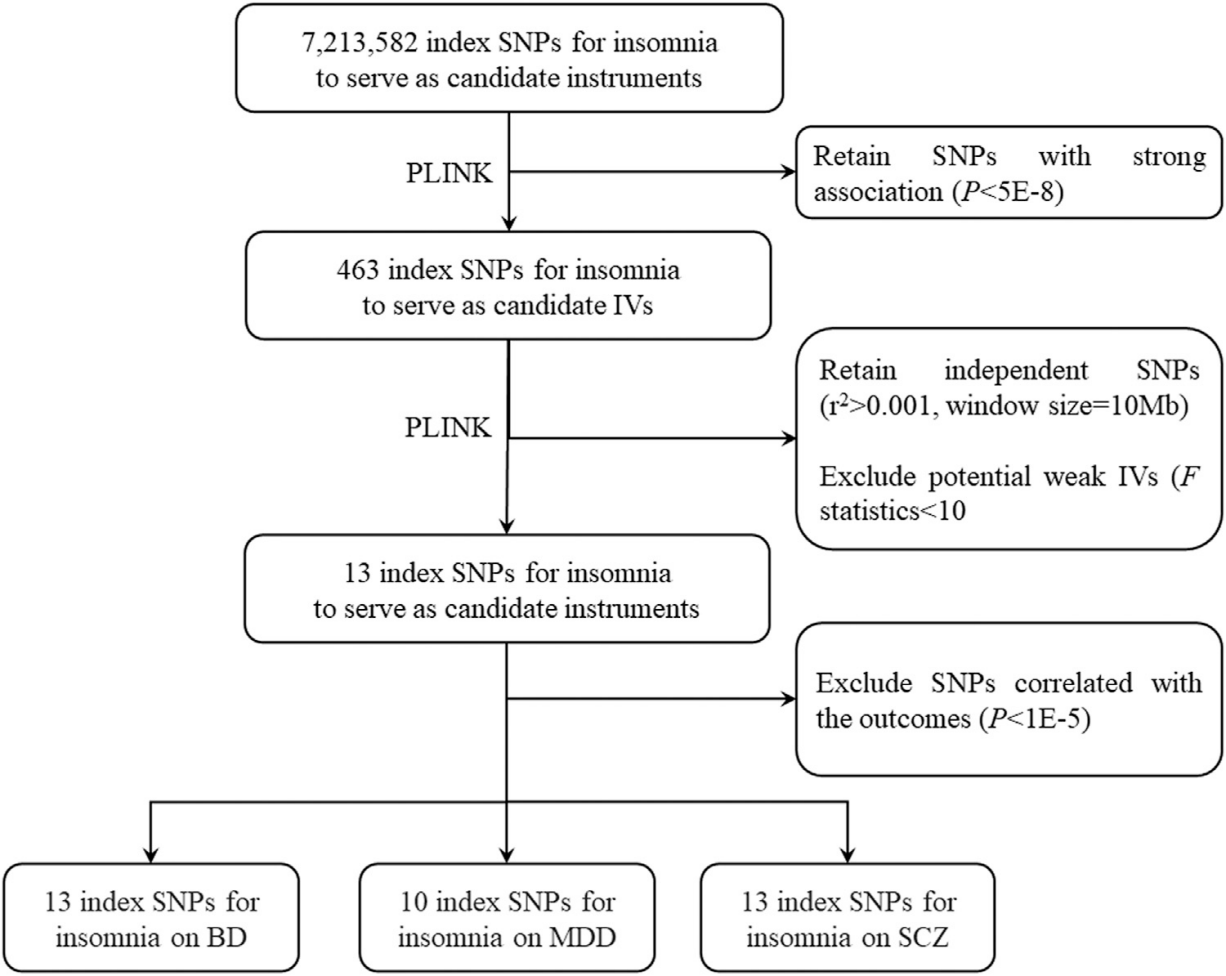
Flow chart for instrumental variable (IV) selection. The flow chart shows the selection process of insomnia IVs to estimate the causal effects on bipolar disorder (BD), major depression (MDD), and schizophrenia (SCZ). First, we use *p* < 5.00E-8 to select index single-nucleotide polymorphisms (SNPs) to ensure that they strongly associate with insomnia. Second, we use *r* ^2^ > 0.001 in the range of 10,000 Mb to select independent index SNPs. We treat the EUR of 1000 Genome Project as the reference panel. The first two steps are completed by PLINK. Finally, we obtain 13 IVs on BD, 10 IVs on MDD, and 13 IVs on SCZ.

Based on the meta-datasets, we followed the strict selection procedure for selecting IVs in other previous MR studies (Zeng et al., 2019; Dong et al., 2021) (Figure 1). First, we retained 463 variants for insomnia with a P-value smaller than 5.00E-8. Second, we excluded 450 highly correlated variants with *r* ^2^ greater than 0.001 in the range of 10 Mb. In addition, following Zeng et al. (2019), we used *F* statistic to test for weak IVs, and no variant was excluded with a minimum *F* statistic of 39.37. Finally, we retained a total of 13 independent candidate IVs for studying the causal relationship between insomnia and BD, MDD, and SCZ. The details are shown in Supplementary Table S2.

We performed three two-sample MR analyses, including inverse variance weighted (IVW), MR-Egger, and weighted median (WM) method, to estimate the potential causal effect of insomnia to BD, MDD, and SCZ (Bowden et al., 2015; Bowden et al., 2016a). Without consideration for the intercept term, IVW regarded the reciprocal of the outcome variance as the weight. Because of no pleiotropy assumption, IVW was biased when pleiotropy exists (Bowden et al., 2015). Differently to IVW, MR-Egger used an intercept term to measure the horizontal pleiotropy between IVs (Bowden et al., 2016b). The weighted median method assumed that variables that account for at least 50% of the total IVs were valid, so the causal effects could be estimated consistently (Bowden et al., 2016a). We also used MR-Egger intercept to test pleiotropy (Hemani et al., 2018; Verbanck et al., 2018; Ong and MacGregor, 2019). All the analyses are performed by R software (v4.1.1). We specially used TwoSampleMR R package (v0.5.6) to perform a MR analysis.

### Sensitivity Analysis

Following Noyce et al. (2017), Zeng et al. (2019), and Zeng and Zhou (2019), we performed a sensitivity analysis to evaluate the potential violations of the model assumptions in the MR analysis: 1) heterogeneity test, 2) pleiotropic test, and 3) LOO sensitivity test. First, heterogeneity analysis estimates heterogeneity between IVs. If heterogeneity existed, it is hardly to direct combinations of IVs. We used the *P*-value of *Q* statistics (*P*_*Q*_) < 0.05 as the significant level. Second, we used MR-PRESSO to test pleiotropy, resulting in serious deviations in MR (Hemani et al., 2018; Ong and MacGregor, 2019). Finally, by gradually excluding each variant, LOO estimated the causal effect of the remaining variants and tested whether the difference between each causal effect is significant. Ideally, defining no significant difference meant a robust result (Noyce et al., 2017). The statistically significant level was set to 0.05.

### Reverse-Direction MR Analyses

We also performed reverse-direction MR to assess the potential reverse causal effects of BD, MDD, and SCZ on insomnia. Following Savage et al. (2018) and Dong et al. (2021), we used the same settings as the abovementioned MR analysis (*p* = 5.00E-8, *r* ^2^ = 0.001, and window size = 10 Mb). We obtained 36 IVs for BD, 44 IVs for MDD, and 50 IVs for SCZ. We used these IVs of three psychiatric disorders to perform reverse causal inferences on insomnia to assess the potential reverse causal effects. The reverse-direction MR analysis process is the same as previously described.

## Results

### Summary of GWAS Meta-Data and Genetic Correlation

We used the meta-analysis datasets to estimate the genetic correlation. The genetic inflation factor (*λ*_gc_) of insomnia is 1.310 (LDSC intercept: 1.015), the *λ*_gc_ of BD is 1.421 (LDSC intercept: 1.080), the *λ*_gc_ of MDD is 1.453 (LDSC intercept: 1.000), and the *λ*_gc_ of SCZ is 1.637 (LDSC intercept: 1.044). The LDSC of the four traits are not larger than 1, which indicates that the meta-datasets are without population stratification. Using GWAS summary statistics to estimate SNP-based observed and liability heritability, these are 0.046 and 0.082 for insomnia, 0.405 and 0.286 for BD, 0.060 and 0.067 for MDD, and 0.295 and 0.170 for SCZ, respectively (Table 1). We use Manhattan plot and qqplot to show the GWAS results for the four traits (Supplementary Figure S1).

**FIGURE 2:**
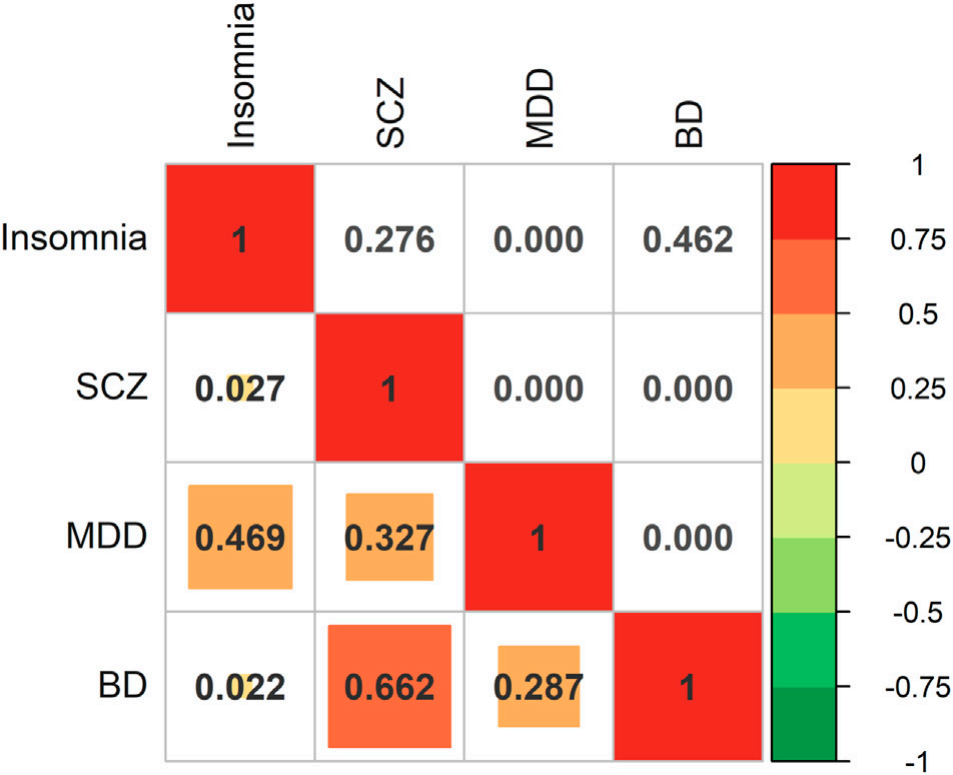
The genetic correlation of four traits using Linkage Disequilibrium SCore regression. The heat map shows the *R*_*g*_ (lower triangle) and its P-value (upper triangle). The red color shows a more positive correlation, while the blue color shows a more negative correlation. We use abbreviations to indicate various psychiatric disorders: BD, bipolar disorder; MDD, major depression; SCZ, schizophrenia.

In addition, we assessed the genetic correlation between BD, MDD, SCZ, and insomnia using cross-trait LDSC. Insomnia was significantly genetically correlated to MDD (*R*_g_ = 0.469, *p *= 2.01E-70), while it was not significantly genetically correlated to BD (*R*_g_ = 0.022, *p* = 0.462) and SCZ (*R*_g_ = 0.027, *p* = 0.276). As expected, we defined three significant genetic correlations between the three psychiatric disorders: genetic correlation between BD and MDD (*R*_g_ = 0.287, *p* = 5.72E-26), between BD and SCZ (*R*_g_ = 0.662, *p* = 5.6E-283), and between MDD and SCZ (*R*_g_ = 0.327, *p* = 4.91E-42) (Figure 2).

### MR Analysis

**FIGURE 3:**
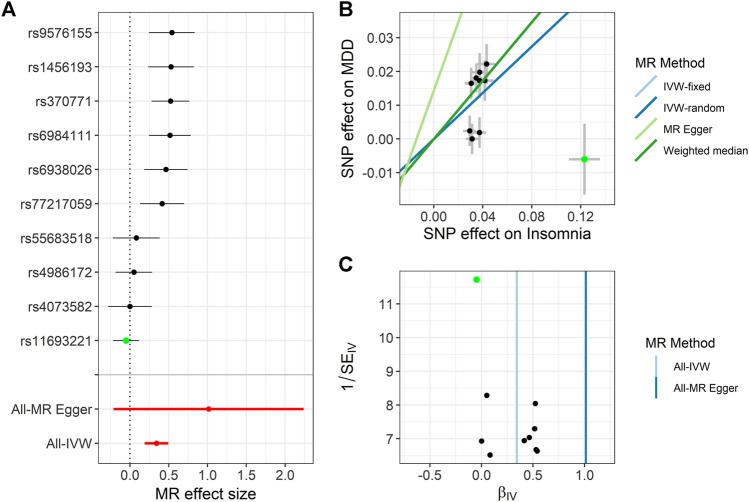
Summary of the Mendelian randomization (MR) analysis for insomnia on major depression (MDD). **(A)** MR effect size of each instrumental variable (IV), MR-Egger, and inverse variance weighted (IVW). **(B)** Scatter plot of causal effects of insomnia on MDD. We use vertical and horizontal black lines to show 95% CI of the estimated effect of IVs on MDD (x-axis) and that on insomnia (y-axis), respectively. We use the blue line to show the IVW random-effects model. The potential SNP outlier (rs11693221) is highlighted in green. **(C)** Funnel plot of the causal effect of insomnia on MDD. Each point represents the estimated causal effect of each IV. The vertical dark blue line represents the causal effect estimate obtained using the MR-Egger method; the light blue line represents the causal effect estimate obtained using the IVW method. The potential outlier (rs11693221) is highlighted in green.

We use the 13 potential IVs of insomnia with the three psychiatric disorders one by one. Specifically, three psychiatric disorders had 13, 10, and 13 IVs, respectively (Supplementary Table S2). Based on different assumptions, we estimate the potential causal effect by all four models, including IVW (fixed- and random-effects model), MR-Egger, and WM. We use the forest plot to show the potential causal effect of the four methods, scatter plot to show the IV effect of insomnia and three psychiatric disorders, and funnel plot to show the relationship between effect of MR model and effect of each SNP (Figure 3, Supplementary Figures S2, S3, Supplementary Tables S3–S5).

**FIGURE 4:**
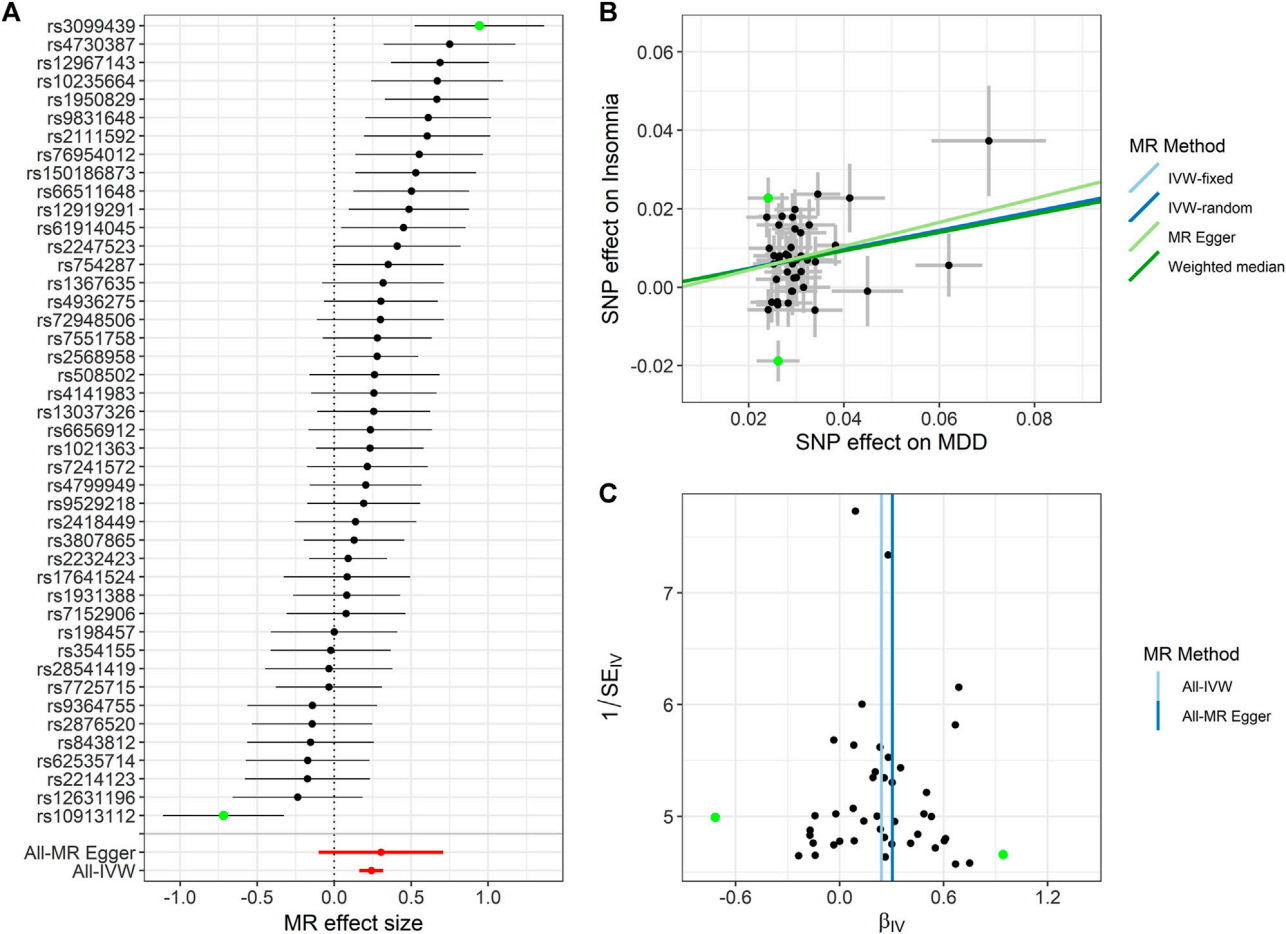
Summary of the reverse-directional Mendelian randomization (MR) analysis for insomnia on major depression (MDD). **(A)** Reverse-directional MR effect size of each instrumental variable (IV), MR-Egger, and inverse variance weighted (IVW). **(B)** Scatter plot of the causal effects of MDD on insomnia. We use vertical and horizontal black lines to show 95% CI of the estimated effect of IVs on MDD (x-axis) and that on insomnia (y-axis), respectively. We use the blue line to show the IVW random-effects model. The potential SNP outliers (rs3099439 and rs10913112) are highlighted in green. **(C)** Funnel plot of the causal effect of MDD on insomnia. Each point represents the estimated causal effect of each IV. The vertical dark blue line represents the causal effect estimate obtained using the MR-Egger method; the light blue line represents the causal effect estimate obtained using the IVW method. The potential outliers (rs3099439 and rs10913112) are highlighted in green.

For MDD, the estimated OR from fixed-effects IVW method is 1.288 (95% CI: 1.189–1.395), with *p* = 5.630E-11. As expected, the result of the random-effects IVW method (OR = 1.288, 95% CI: 1.091–1.520, *p* = 0.003) is similar to that of the random-effects IVW. However, the result of WM (OR = 1.076, 95% CI: 0.915–1.216, *p* = 0.374) and MR-Egger (OR = 0.916, 95% CI: 0.599–1.401, *p* = 0.696) is not similar to that of IVW (Figure 4, Supplementary Table S4). The abovementioned results indicate that the risk of MDD increases with the increasing level of insomnia. We should use the result of the sensitivity analysis to determine which one is the main result.

For BD, in terms of the fixed-effects IVW method, the estimated OR of insomnia is 1.216 (95% CI: 0.974–1.518, *p* = 0.084). As expected, the result of the random-effects IVW method is similar to that of the fixed-effects method, with OR = 1.216 (95% CI: 0.801–1.845) and *p* = 0.358. The results of WM (OR = 1.351, 95% CI: 0.948–1.917, *p* = 0.096) and MR-Egger (OR = 1.909, 95% CI: 0.506–7.197, *p* = 0.360) are similar (Supplementary Figure S2, Supplementary Table S3). Unfortunately, the results of all MR methods are not significant, suggesting that there might be no potential causal association for insomnia on BD. The specific results have to be verified after a sensitivity analysis.

Finally, for SCZ, the estimated OR of insomnia by the fixed-effects IVW method is 0.787 (95% CI: 0.630–0.983, *p* = 0.035), while the OR from the random-effect model is 0.787 (95% CI: 0.479–1.292, *p* = 0.344). In addition, the results of the weighted median method (OR = 0.604, 95% CI: 0.413–0.883, *p* = 0.009) and MR-Egger (OR = 0.566, 95% CI: 0.109–2.950, *p* = 0.513) are different (Supplementary Figure S3, Supplementary Table S5). Similarly, which specific result is representative also needs to be determined after the sensitivity analysis.

### Sensitivity Analyses

Using three kinds of MR methods, we identify the potential causal relationship of insomnia on MDD (IVW method) and SCZ (only WM method). We performed a series of sensitivity analyses to assess whether the results obtained are robust, whether there is potential bias (such as pleiotropy and data heterogeneity), and whether there is a certain IV that seriously affects the outcome variable.

First, we conducted a heterogeneity analysis. Based on IVW, the *P*_*Q*_ values of BD, MDD, and SCZ are 2.83E-5, 1.39E-5, and 2.4E-8, respectively. Following Zeng et al. (2019), we selected the result from the random-effects model or deleted SNPs with *P*-value < 1.00E-5. Because of the similarity between the fix- and random-effects IVW methods, we deleted SNPs with *P*-value < 1.00E-5 to reduce heterogeneity. For BD, excluding rs6938026, the heterogeneity (*P*_*Q*_ = 0.010) is reduced. For MDD, the heterogeneity (*P*_*Q*_ = 0.004) is reduced after excluding rs11693221. The heterogeneity of SCZ (*P*_*Q*_ = 0.078) is also reduced after excluding rs6938026 and rs370771.

In addition, we performed a series of pleiotropic tests to further ensure the validation of MR analysis. For MDD, MR-Egger showed that the intercept is not statistically significant (*p* = 0.131), which indicated that there was no horizontal pleiotropy that existed among IVs. The MR-PRESSO outlier test suggested that rs11693221 (RSS_obs_ = 2.32E-3, *p* < 0.01) was a potential outlier. We used the MR-PRESSO distortion test and LOO test to test whether the causal effect changed with or without the outlier, but their results were different (*P*_MR-PRESSO_ = 0.282 and *P*_LOO_ = 1.03E-05). Then, even excluding the outlier, the heterogeneity test showed statistically significant heterogeneity (*P*_*Q*_ = 0.004). Therefore, we used the result from random-effects IVW method with the outlier excluded to represent the casual effect of insomnia on MDD (OR = 1.408, 95%CI: 1.209–1.640, *p* = 1.03E-05) (Figure 3 and Supplementary Table S4).

For BD, MR-Egger showed that the intercept is not statistically significant (*p* = 0.496). The MR-PRESSO outlier test suggested that rs6938026 (RSS_obs_ = 2.89E-3, *p* < 0.01) and rs77960 (RSS_obs_ = 2.28E-3, *p* = 0.013) were the potential outliers. However, the MR-PRESSO distortion test and LOO test indicated that no statistical significance could be identified when excluding the two variants (*P*_MR-PRESSO_ = 0.979, *P*_LOO1_ = 0.147, and *P*_LOO1_ = 0.622). Though heterogeneity was reduced without the two outliers, we fail to define a statistically significant causal effect for insomnia on BD (Supplementary Figure S2 and Supplementary Table S3).

For SCZ, MR-Egger showed that the intercept is not statistically significant (*p* = 0.689). The MR-PRESSO outlier test indicated that four SNPs, including rs1456193, rs370771, rs4986172, and rs6938026, were identified as potential outliers. However, the MR-PRESSO distortion test and LOO test indicated that no statistical significance could be identified when excluding the two variants (*P*_MR-PRESSO_ = 0.738, *P*_LOO1_ = 0.172, *P*_LOO2_ = 0.588, *P*_LOO3_ = 0.096, and *P*_LOO4_ = 0.613). Though there was no significant heterogeneity between models with and without outliers, we used the result from the random-effects IVW method with the outliers excluded to represent the casual effect of insomnia on SCZ for caution (OR = 0.752, 95%CI: 0.524–1.079, *p =* 0.122) (Supplementary Figure S3 and Supplementary Table S5).

### Reverse-Direction MR Analysis

Following a previous MR analysis (Hartwig et al., 2017; Dong et al., 2021), in order to identify the potential confounding factors that mislead the direction of causal effects, we performed reverse-direction MR (Figure 4, Supplementary Figures S4, S5). We found that MDD and SCZ have a significantly potential causal association to insomnia, while a potential causal effect for BD on insomnia is not significant. Specifically, using IVW, the estimated OR for MDD and SCZ on insomnia is 1.273 (*p* = 1.097E-9) and 1.028 (*p* = 0.004), respectively (Figure 4 and Supplementary Figure S4). The results indicate that the risk of BD and SCZ could increase the risk of insomnia.

## Discussion

Using the summary statistics of four traits and reference LD panel from public sources, we performed a two-sample MR analysis to show the causal effects of insomnia on three psychiatric disorders. We found that the causal OR of insomnia on MDD is 1.288, that the reverse direction causal OR of MDD on insomnia is 1.230, and that no statistical significance is defined for insomnia on BD and SCZ. These results were based on several MR methods to guard against potential model misspecifications and is consistent in the estimates of causal effects, suggesting that the findings are convincing.

As we have known, many observational studies aim to explore the associations between insomnia and BD, MDD, and SCZ. A case–control study found that insomnia significantly affected patients with BP with depressive symptoms (OR = 4.17, *p* = 0.043), and sleep disturbances also predicted manic symptoms (OR = 8.69, *p* = 0.001) (Palagini et al., 2020). Integrating 21 observational studies for insomnia on DP, Baglioni *et al.* show that the overall OR of insomnia is 2.60 (Baglioni et al., 2011). A cross-sectional study found that the effect size of insomnia-caused symptoms of depression or anxiety is 3.01 (Batalla-Martín et al., 2020). The abovementioned studies have shown that insomnia is a risk factor to psychiatric disorders. Differently to previous studies, the effect size from MR is directional.

The causal relationship between insomnia and BD, MDD, and SCZ identified in the European population was estimated using the IVs of insomnia in three different outcomes. However, we also recognize that there is still a large amount of unexplainable diversity in the etiology of BD, MDD, and SCZ in European populations. Further research is needed to understand the genetic and environmental factors behind the differences between BD, MDD, and SCZ. Although many studies have confirmed the potential impact of insomnia symptoms on some psychiatric disorders, as mentioned earlier, there is no clear answer yet, and it is not clear whether insomnia has a significant causal effect on these diseases.

Like other MR analyses, our results are not without any drawbacks. First, MR cannot completely exclude all confounding factors because the relationship between exposure and outcome obtained through the observational data used in MR analysis is not a pure relationship between exposure and outcome (Sekula et al., 2016; Ference et al., 2019). In our research, it may be because the sample size of BD and SCZ is relatively small compared with insomnia, and the effect of exposure on the results is relatively weak. The statistical power of MR analysis for certain exposures is limited, resulting in negative results (Pierce and Burgess, 2013). Second, we defined the bidirectional causal association between insomnia and MDD. This plays an important supplement to support the causal association, such that it is hard to detangle the relationship between them using either a cross-sectional study or a MR analysis (Manber and Chambers, 2009; Fang et al., 2019). Nevertheless, our study also provides help for new developments in psychiatric disorder research and new treatment strategies in the future (Hertenstein et al., 2019).

## Conclusion

The result of the MR and additional analyses shows that insomnia has a positive causal effect on MDD in the European population and provides new evidence of the causal relationship with insomnia on BD and SCZ in European populations.

## Data Availability

The original contributions presented in the study are included in the article/Supplementary Material, further inquiries can be directed to the corresponding author.
