# Supplementary material for: The Causal Effects of Insomnia on Bipolar Disorder, Depression, and Schizophrenia: A Two-Sample Mendelian Randomization Study
Source: Front Genet. 2021 Oct 11;12:763259. doi: 10.3389/fgene.2021.763259 (PMC8542855; doi:10.3389/fgene.2021.763259)
Supplement: Supplementary file 1 [file DataSheet2.docx]

Supplementary Material

## Supplementary Figures


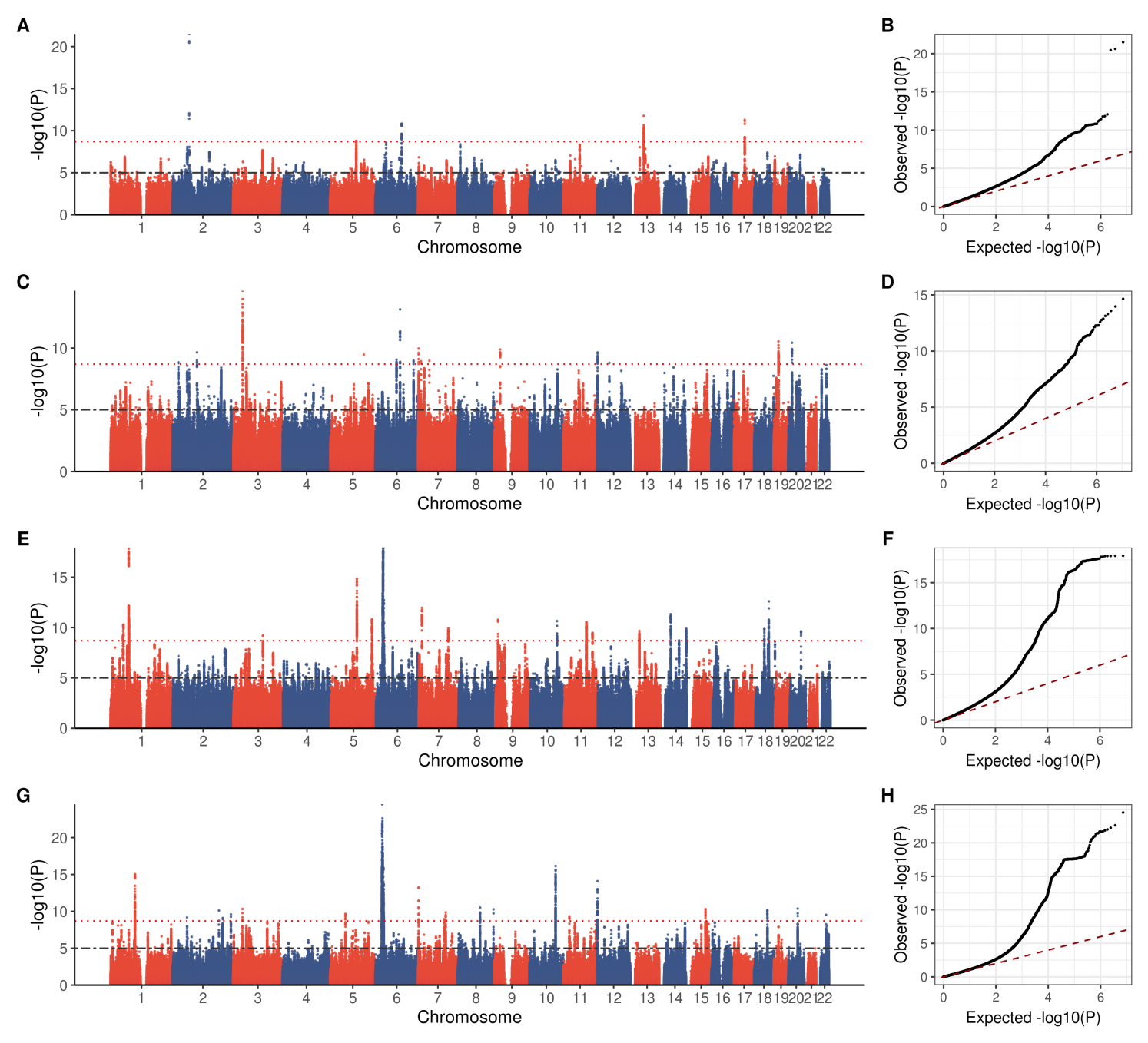


**Supplementary Figure S1.** Manhattan plot and qqplot show GWAS results of four traits. **(A)** and **(B)** are the Manhattan plot and qqplot for insomnia, respectively; **(C)** and **(D)** are for BD; **(E)** and **(F)** are for MDD; **(G)** and (**H)** are for SCZ.

**
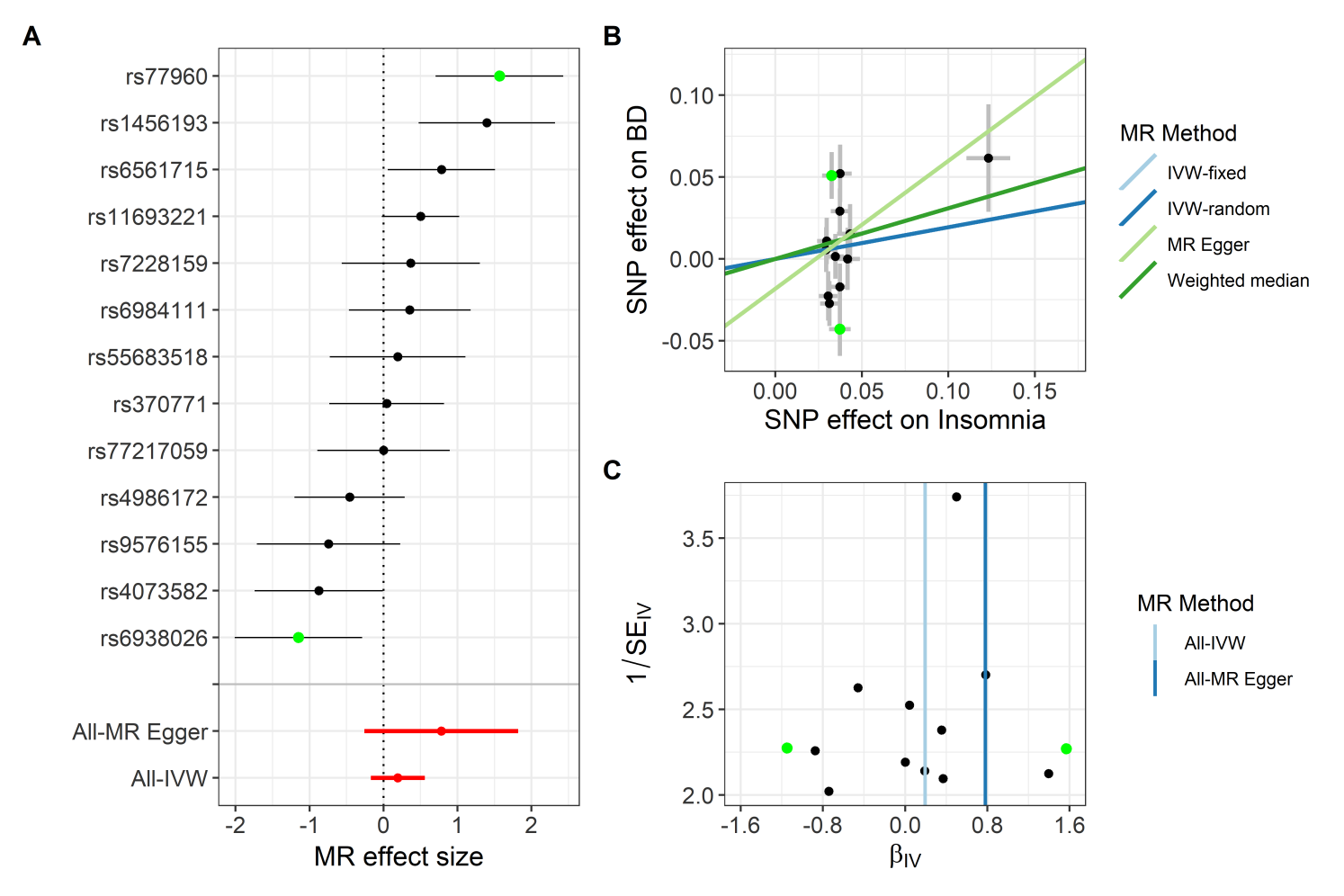
Supplementary Figure S2.** **Summary of the MR analysis for insomnia on BD**. **(A)** The MR effect size of each IV, MR-Egger and IVW. **(B)** The scatter plot of causal effects of insomnia on BD. We use vertical and horizontal black line to show 95% CI of the estimated effect of IVs on BD (x-axis) and that on insomnia (y-axis), respectively. We use the bule line to show the IVW random-effects model. Potential SNP outliers (rs77960 and rs6938026) are highlighted in green. **(C)** The funnel plot of the causal effect of insomnia on BD. Each point represents the estimated causal effect of each IV. The vertical dark blue line represents the causal effect estimate obtained using the MR-Egger method; the light blue line represents the causal effect estimate obtained using the IVW method. Potential outliers (rs77960 and rs6938026) are highlighted in green.

**
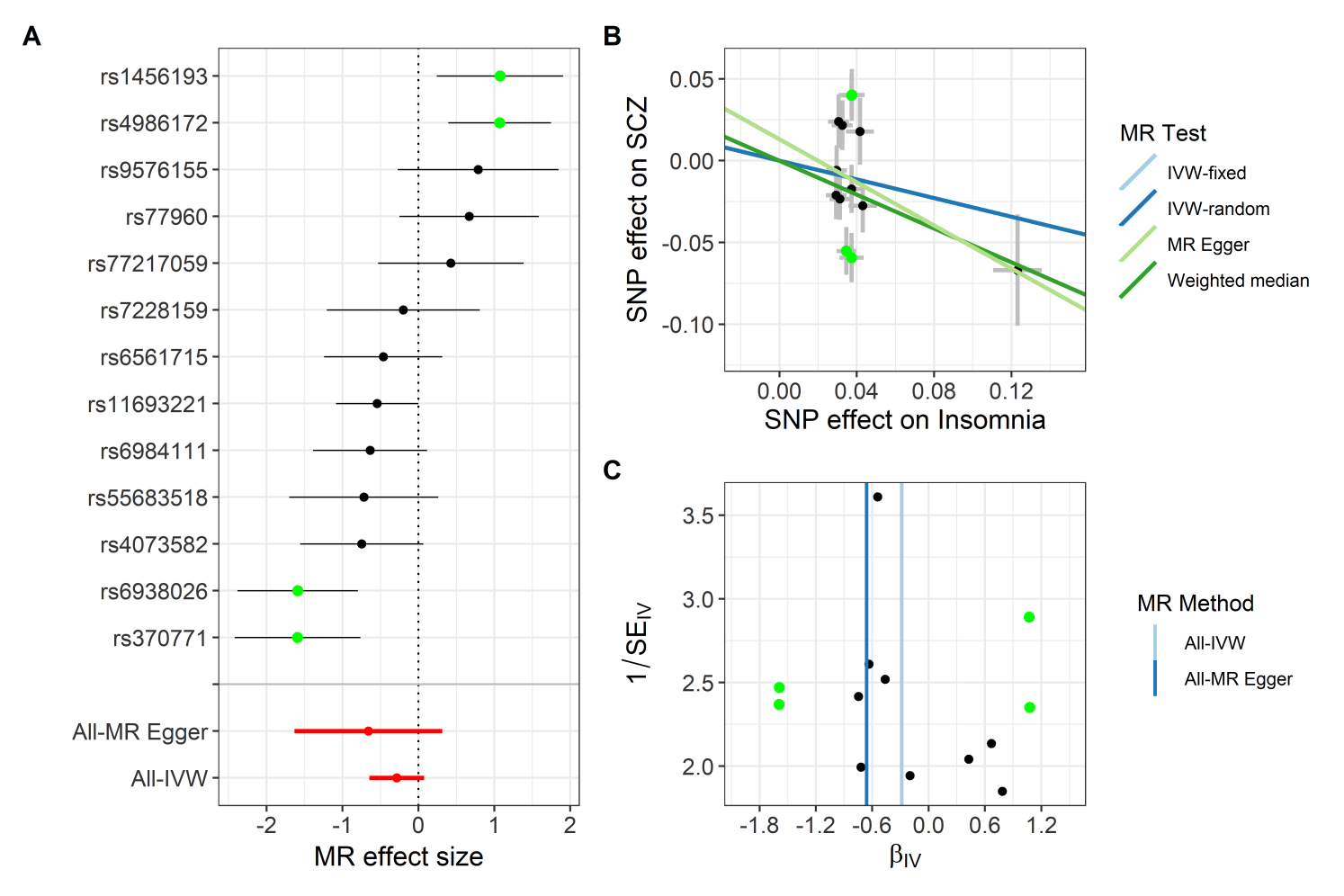
**

**Supplementary Figure S3.** **Summary of the MR analysis for insomnia on SCZ (A)** The MR effect size of each IV, MR-Egger and IVW. **(B)** The scatter plot of causal effects of insomnia on SCZ. We use vertical and horizontal black line to show 95% CI of the estimated effect of IVs on insomnia (x-axis) and that on SCZ (y-axis), respectively. We use the bule line to show the IVW random-effects model. Potential SNP outliers (rs1456193, rs4986172, rs6938026 and rs370771) are highlighted in green. **(C)** The funnel plot of the causal effect of insomnia on SCZ. Each point represents the estimated causal effect of each IV. The vertical dark blue line represents the causal effect estimate obtained using the MR-Egger method; the light blue line represents the causal effect estimate obtained using the IVW method. Potential outliers (rs1456193, rs4986172, rs6938026 and rs370771) are highlighted in green.


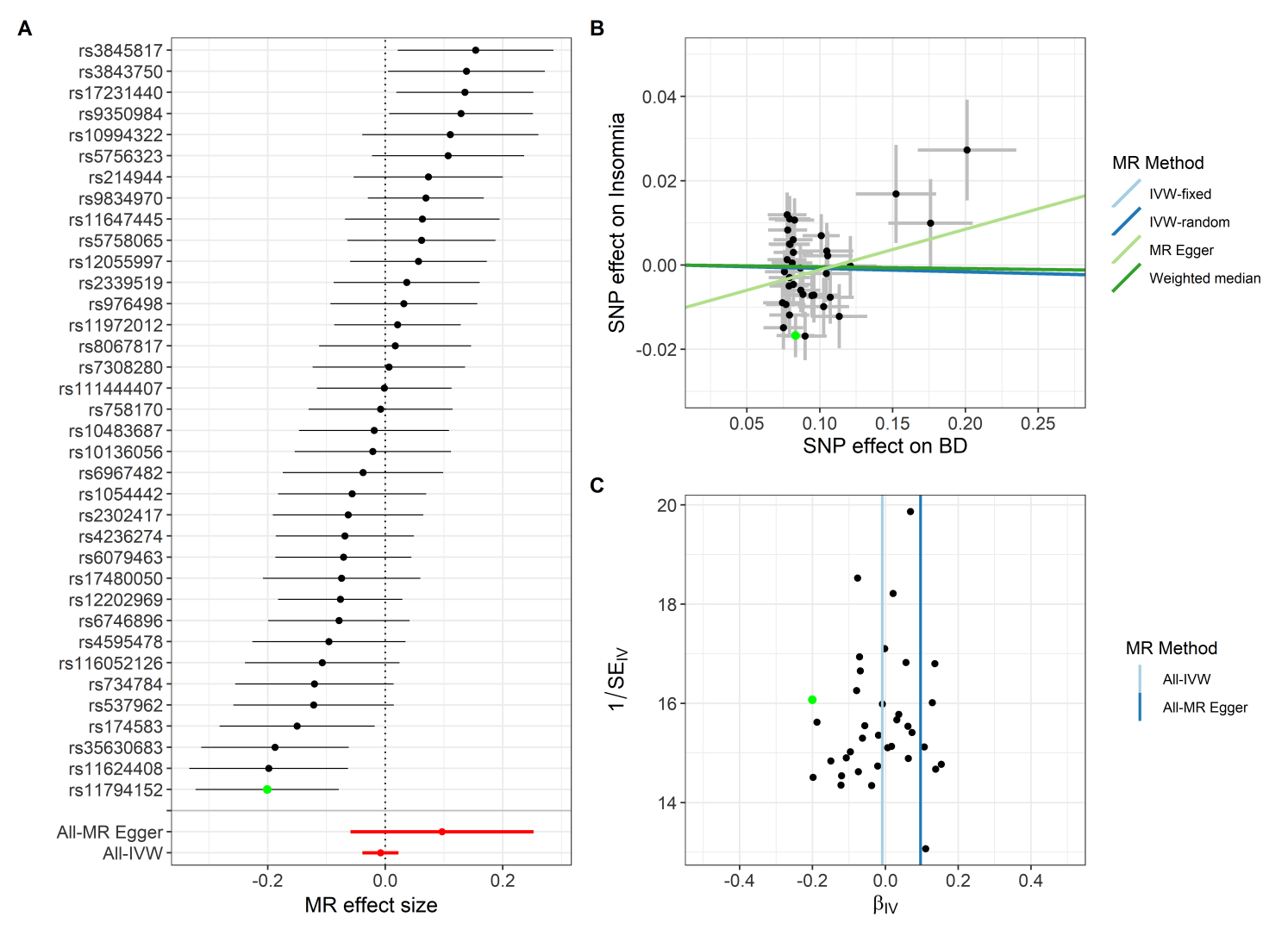


**Supplementary Figure S4.** **Summary of the reverse-directional MR analysis for insomnia on BD**. **(A)** The reverse-directional MR effect size of each IV, MR-Egger and IVW. **(B)** The scatter plot of causal effects of BD on insomnia. We use vertical and horizontal black line to show 95% CI of the estimated effect of IVs on BD (x-axis) and that on insomnia (y-axis), respectively. We use the bule line to show the IVW random-effects model. Potential SNP outlier (rs11794152) is highlighted in green. **(C)** The funnel plot of the causal effect of BD on insomnia. Each point represents the estimated causal effect of each IV. The vertical dark blue line represents the causal effect estimate obtained using the MR-Egger method; the light blue line represents the causal effect estimate obtained using the IVW method. Potential SNP outlier (rs11794152) is highlighted in green.


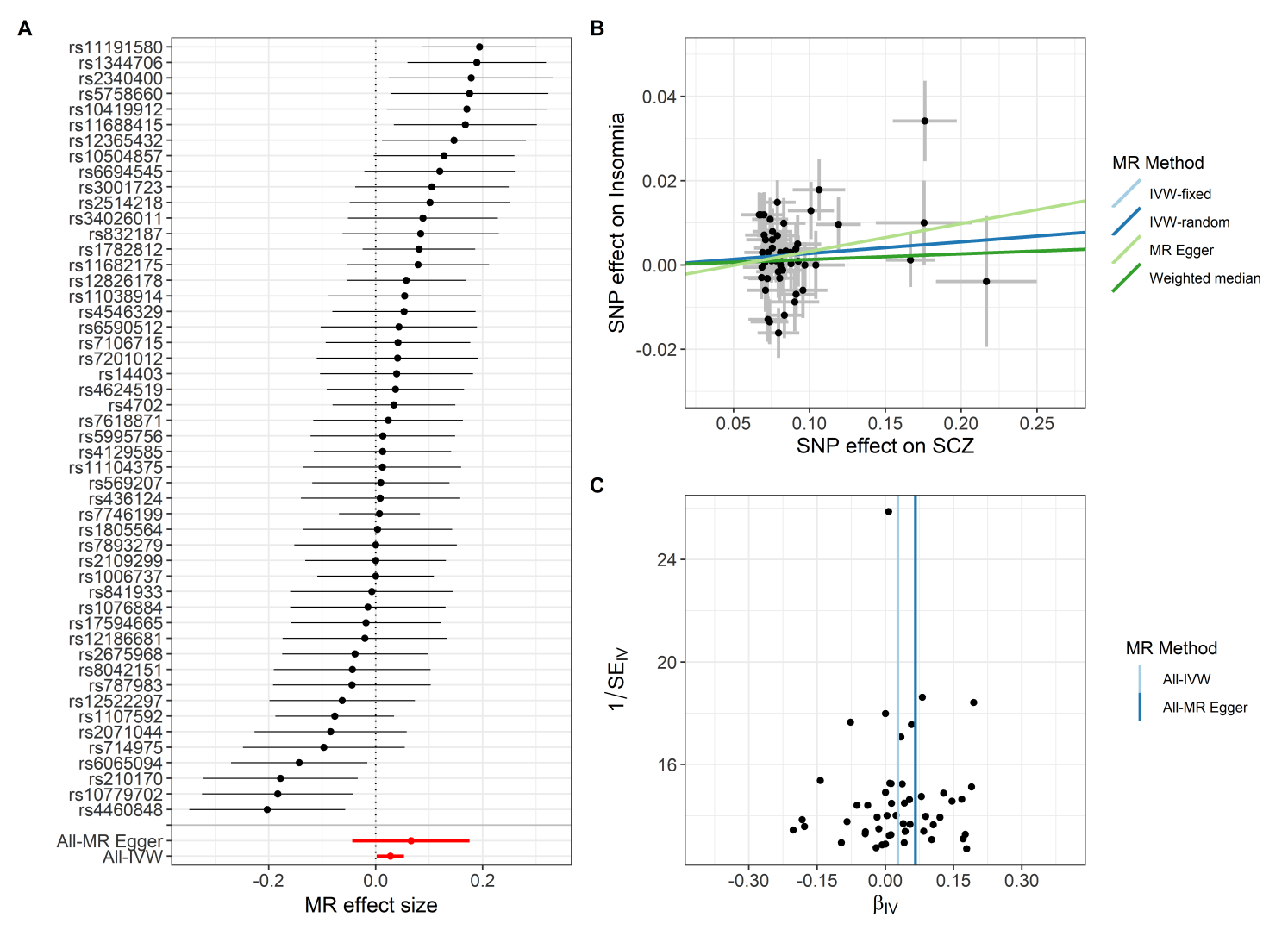


**Supplementary Figure S5.** **Summary of the reverse-directional MR analysis for insomnia on SCZ**. **(A)** The reverse-directional MR effect size of each IV, MR-Egger and IVW. **(B)** The scatter plot of causal effects of SCZ on insomnia. We use vertical and horizontal black line to show 95% CI of the estimated effect of IVs on SCZ (x-axis) and that on insomnia (y-axis), respectively. We use the bule line to show the IVW random-effects model. **(C)** The funnel plot of the causal effect of MDD on insomnia. Each point represents the estimated causal effect of each IV. The vertical dark blue line represents the causal effect estimate obtained using the MR-Egger method; the light blue line represents the causal effect estimate obtained using the IVW method.
